# Long non‐coding RNA NEAT1 promotes aerobic glycolysis and progression of cervical cancer through WNT/β‐catenin/PDK1 axis

**DOI:** 10.1002/cam4.7221

**Published:** 2024-05-11

**Authors:** Min Su, Ziyan Liang, Shidong Shan, Yang Gao, Li He, Xuelian Liu, Anjin Wang, Hua Wang, Hongbing Cai

**Affiliations:** ^1^ Department of Gynecological Oncology, Zhongnan Hospital Wuhan University Wuhan People's Republic of China; ^2^ Hubei Key Laboratory of Tumor Biological Behaviors Wuhan People's Republic of China; ^3^ Hubei Cancer Clinical Study Center Wuhan People's Republic of China; ^4^ Department of Urology, Zhongnan Hospital Wuhan University Wuhan People's Republic of China; ^5^ Department of Radiation and Medical Oncology, Zhongnan Hospital Wuhan University Wuhan People's Republic of China

**Keywords:** cervical cancer, glycolysis, NEAT1, PDK1, WNT/β‐catenin

## Abstract

**Background:**

Cervical cancer is one of the most common gynecological cancers. Accumulated evidence shows that long non‐coding RNAs (lncRNAs) play essential roles in cervical cancer occurrence and progression, but their specific functions and mechanisms remain to be further explored.

**Methods:**

The RT‐qPCR assay was used to detect the expression of NEAT1 in cervical cancer tissues and cell lines. CCK‐8, colony formation, flow cytometry, western blotting, and Transwell assays were used to evaluate the impact of NEAT1 on the malignant behavior of cervical cancer cells. Glucose consumption, lactate production, ATP levels, ROS levels, MMP levels, and the mRNA expressions of glycolysis‐related genes and tricarboxylic acid cycle‐related genes were detected to analyze the effect of NEAT1 on metabolism reprograming in cervical cancer cells. The expressions of PDK1, β‐catenin and downstream molecules of the WNT/β‐catenin signaling pathway in cervical cancer cells and tissues were detected by western blotting, RT‐qPCR, immunofluorescence and immunohistochemistry assays.

**Results:**

This study investigated the role and possible molecular mechanism of lncRNA nuclear paraspeckle assembly transcript 1 (NEAT1) in cervical cancer. Our results showed that NEAT1 was highly expressed in cervical cancer tissues and cell lines. Downregulation of NEAT1 inhibited the proliferation, migration, invasion and glycolysis of cervical cancer cells, while overexpression of NEAT1 led to the opposite effects. Mechanistically, NEAT1 upregulated pyruvate dehydrogenase kinase (PDK1) through the WNT/β‐catenin signaling pathway, which enhanced glycolysis and then facilitated cervical cancer metastasis. Furthermore, NEAT1 maintained the protein stability of β‐catenin but did not affect its mRNA level. We also excluded the direct binding of NEAT1 to the β‐catenin protein via RNA pull‐down assay. The suppressive impact of NEAT1 knockdown on cell proliferation, invasion, and migration was rescued by β‐catenin overexpression. The WNT inhibitor iCRT3 attenuated the carcinogenic effect induced by NEAT1 overexpression.

**Conclusion:**

In summary, these findings indicated that NEAT1 may contribute to the progression of cervical cancer by activating the WNT/β‐catenin/PDK1 signaling axis.

## Graphical abstract

In this article, we focused on the essential role and specific mechanism of NEAT1 in metabolism reprograming. We highlighted the vital role of WNT/β‐catenin pathway in the regulation of aerobic glycolysis through targeting the key enzyme PDK1, which not only accelerated aerobic glycolysis but also damaged the function of mitochondrion to a certain extent and thus induced the conversion of glucose metabolism. NEAT1 slowed down the degradation of β‐catenin and thereby increased the nuclear accumulation of β‐catenin to activate WNT/β‐catenin signaling even which had no direct interaction with β‐catenin.

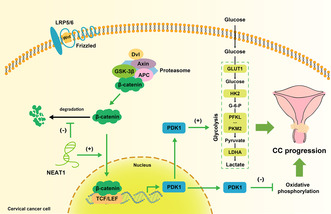

## 1 INTRODUCTION

In developing countries, cervical cancer remains a major threat to women, despite HPV vaccination, primary screening, and aggressive treatment.^1^, ^2^ Despite the gradual development of targeted therapy and immunotherapy, metastasis, and recurrence remain the major causes of death in patients with cervical cancer.^3^ Thus, it is crucial to identify the underlying molecular mechanism of recurrence and metastasis and potential therapeutic targets for improving the prognosis of cervical cancer patients.

Long non‐coding RNAs (LncRNAs) are transcripts longer than 200 nucleotides that can act as tumor promoters or tumor suppressors in the progression of malignant cancer.^4^, ^5^, ^6^, ^7^, ^8^ The lncRNA NEAT1 is the essential component of paraspeckle,^9^ which is abnormally expressed and participates in the progression, metastasis and poor prognosis of malignant cancer^10^ in lung cancer,^11^ laryngeal cancer,^12^ colorectal cancer,^13^ hepatocellular carcinoma,^14^ and gastric cancer.^15^, ^16^ Given the crucial role of NEAT1 in cancer, identifying the function and underlying mechanism of NEAT1 in the metastasis of cervical cancer and investigating its therapeutic potential are highly important.

The present study investigated a novel mechanism through which NEAT1 contributed to cervical cancer metastasis and malignant progression. Our in vitro study demonstrated that NEAT1 was highly expressed in cervical cancer, promoting the growth, invasion, and migration of cervical cancer cells. Mechanistically, NEAT1 modulated WNT/β‐catenin/PDK1 axis to facilitate aerobic glycolysis, resulting in EMT and metastasis in cervical cancer. In general, our findings suggested that NEAT1 functioned as an oncogene and that targeting NEAT1 could be a potential therapeutic avenue in cervical cancer.

## 2 MATERIALS AND METHODS

### 2.1 Human cervical cancer tissue specimens

Forty‐four pathologically confirmed cervical cancer tissue specimens and 22 normal cervical tissue specimens were collected from recent patients at the Affiliated Zhongnan Hospital of Wuhan University. All human samples were obtained with the approval of the Medical Ethics Committee of Zhongnan Hospital of Wuhan University (No. 2021087), and written informed consent was obtained from all patients or their relatives. All human studies were carried out in accordance with the principles of the Declaration of Helsinki.

### 2.2 Cell culture and transfection

The human‐derived cervical cancer cell lines HeLa (RRID: CVCL_0030) and SiHa (RRID: CVCL_0032) were purchased from the American Type Culture Collection (ATCC, USA). Both cell lines were cultured in a 37°C humidified incubator with CO_2_ using high‐glucose DMEM (Gibco, USA) supplemented with 10% FBS (Gibco, USA). 2‐DG and LiCl were obtained from Solarbio (D8930, C8380, CHN), while iCTR3 was obtained from MedChemExpress (MCE, HY‐103705, USA). A STR profiling test was performed on all cell lines in order to confirm their mycoplasma‐free status. Two siRNAs targeting NEAT1 (si‐NEAT1#1: 5′‐GCCTTGTAGATGGAGCTTGC‐3′, si‐NEAT1#2: 5′‐GUGAGAAGUUGCUUAGAAAUU‐3′) and one siRNA targeting PDK1 (si‐PDK1: 5′‐GGUUGUUGUUGGAGAAGCATT‐3′) were purchased from GenePharma (CHN). The pcDNA3.1 vector, pEGFP vector, pcDNA3.1‐NEAT1 plasmid and pEGFP‐CTNNB1 plasmid were purchased from Tsingke (Beijing, CHN). These siRNAs or plasmids were transfected into cells using Lipofectamine 2000 (Invitrogen, USA).

### 2.3 Cell Counting Kit‐8 assay

Two thousand cells per well were seeded into 96‐well plates and incubated at 37°C. Ten microliters of cell counting kit‐8 (CCK‐8, Meilune, CHN) reagent was added to each well every 24 h. After incubating for 1 h, measuring the absorbance at 450 nm to determine the cell proliferative ability.

### 2.4 Flow cytometry analysis of the cell cycle

Each group of cells was collected and washed twice using ice‐cold PBS and then mixed with 1 mL of DNA staining solution and 10 μL of permeabilization solution (Multi Sciences, CHN). Samples were then incubated in the dark for 30 min. Flow cytometry was used to detect the cell cycle.

### 2.5 Colony formation assay

For the colony formation assay, 300 cells were seeded into each well of a 6‐well plate and cultured for 2–3 weeks. Every 2–3 days, the medium was changed. Cells were fixed with 4% paraformaldehyde, stained with 0.5% crystal violet for 30 min, and then washed with cold PBS when there were more than 50 cells in each colony. The inverted plates were photographed and the number of colonies was counted.

### 2.6 Transwell migration and invasion assays

Transwell migration assay: 3 × 10^4^ cells were seeded into each chamber (Corning, 8‐μm pore size, USA). Then, 150 μL of FBS‐free DMEM and 700 μL of DMEM supplemented with 20% FBS were added to the upper and lower chambers, respectively. The migrating cells were then fixed, stained and observed under a microscope after they were cultured for 48 h.

Transwell invasion assay: Matrigel gel (BD Biocoat, USA) was diluted 8‐fold with FBS‐free medium. And 50 μL of diluted Matrigel was added to cover the bottom of the chamber. The remaining steps were the same as above.

### 2.7 Analysis of glucose consumption, lactate production, and ATP levels

Glucose consumption, lactate production and ATP levels was detected by glucose assay kit (glucose oxidase method, Jiancheng Bio, CHN), lactate acid assay kit (KeyGEN Bio, CHN) and ATP assay kit (Beyotime, CHN), respectively, according to the manufacturer's protocols. All values were normalized to the cell number or total protein levels.

### 2.8 Reactive oxygen species and mitochondrial membrane potential measurements

Cells in 6‐well plates were treated with DCFH‐DA (10 μM, Beyotime, CHN), which was diluted with serum‐free DMEM. The cells were incubated in the incubator for 20 min in the dark and then washed with serum‐free medium three times. In addition, the prepared JC‐1 staining solution (Beyotime, CHN) was used to treat the cells or the cell sediments, and JC‐1 staining buffer was used to wash them. Flow cytometry and inverted fluorescence microscopy (Olympus Corporation, JPN) were applied to examine the ROS levels and changes in MMP.

### 2.9 mRNA stability assay

Stability of RNA was achieved by treating cells with actinomycin D (Act‐D, 5 μM, MCE, CHN). Then, the cells were collected at the indicated times, and RNA was isolated. The half‐life (t1/2) of CTNNB1 mRNA was calculated, and ACTB was used for normalization.

### 2.10 Protein stability assay

The cells in each group were treated with cycloheximide (CHX, 20 μg/mL Solarbio, CHN). Total protein was extracted at 0 (without CHX), 2, 4, 8, and 12 h after treatment, and protein expression was detected through western blotting analysis.

### 2.11 RNA pull‐down assay

T7‐Flash biotin‐RNA transcription reactions were performed using a T7 biotin‐labeled RNA synthesis kit (GENESEED, CHN) to prepare the RNA probe. Following the manufacturer's protocol of RNA–protein pull‐down kit (GENESEED, CHN), 1 × 10^7^ HeLa cells were collected and resuspended in 1 mL of capture buffer, to which 10 μL of RNase inhibitor and 10 μL of proteinase inhibitor were added. Then, 50 μL of streptavidin magnetic beads was incubated with 50 pmol of biotin‐labeled RNA probes for 30 min and then mixed with the cell lysates, rotating at 4°C for 1 h. After washing the beads with wash buffer, 5× SDS–PAGE loading buffer was added. Western blotting was performed to detect the products.

### 2.12 Cell immunofluorescence staining

Cells were seeded into 6‐well plates in which 14‐mm cell climbing slices had settled. Cells were grown to a suitable density, washed with ice‐cold PBS, fixed with 4% paraformaldehyde, washed with PBS three times, permeabilized with Triton X‐100 (Biosharp, CHN), and blocked with goat serum at room temperature in sequence. The slices were incubated with primary antibodies for 2 h at room temperature and then with Cy3‐labeled or FITC‐labeled secondary antibodies for 1 h. DAPI was used to stain the cell nucleus. A fluorescence microscope was used to capture the images.

### 2.13 Reverse transcription quantitative polymerase chain reaction (RT–qPCR)

Total RNA was extracted using TRIzol Reagent (Invitrogen, USA), and the concentration and quality of the RNA were measured with a NanoPhotometer spectrophotometer (Thermo, USA). A Hiscript II Q Select RT kit (Vazyme, CHN) was used for RNA reverse transcription. PCR was performed using the iTaqTM Universal SYBR Green Supermix (Vazyme, CHN). The expression levels of RNA were analyzed via the 2^−ΔΔCt^ method. The sequences of primers used in this study are listed in Table S1.

### 2.14 Isolation and extraction of nuclear and cytoplasmic proteins

Nuclear and Cytoplasmic Protein Extraction Kit (Beyotime, CHN) was used to isolate the nuclear and cytoplasmic proteins following the instructions. Reagent A containing PMSF (Biosharp, CHN) was added to lyse the cells for 15 min. After adding 10 μL of reagent B, the mixtures were vortexed at high speed in an ice bath and then centrifuged at low temperature to obtain the cytoplasmic protein. For nuclear protein extraction, 50 μL of nuclear protein extraction reagent was added to the precipitate, followed by intermittent vortexing and incubation in an ice bath for 30 min. Lamin B and β‐actin were used as internal controls for the nuclear and cytoplasmic fractions, respectively.

### 2.15 Western blotting analysis

Total protein was extracted through lysis with RIPA (Radio Immunoprecipitation Assay, Biosharp, CHN) reagent containing PMSF and phosphatase inhibitors for 30 min, followed by centrifugation to collect the supernatant. BCA protein concentration assay kit (Beyotime, CHN) was used to measure the protein concentration. Thirty micrograms of protein was separated by SDS–PAGE and then transferred onto PVDF membranes (Millipore, USA). Membranes were incubated overnight with primary antibodies at 4°C after blocking with 5% skim milk. The membranes were then incubated with secondary antibodies for 1 h at room temperature. A chemiluminescence kit (Thermo Fisher Scientific, USA) was used to visualize the proteins. ImageJ software (National Institutes of Health, USA) was used to quantify the images. The antibodies used in this study are listed in Table S2.

### 2.16 Immunohistochemistry

The cervical cancer slides were incubated at 60°C for 2 h and then deparaffinized and hydrated with dimethylbenzene and ethanol. Citrate buffer was used to reverse the antigens, and 3% H_2_O_2_ was used to block endogenous peroxidase activity. The slides were incubated with primary antibodies overnight at 4°C after blocking with bovine serum albumin (BSA). Then, the slides were incubated with secondary antibodies. The slides were stained with dimethylbenzene and hematoxylin and then sealed with neutral gum. All the tissue samples were scanned under a fluorescence microscope.

### 2.17 Statistical analysis

The data are showed as the mean ± standard deviation (SD). Three independent experiments was performed. Comparisons between groups was performed by Student's *t*‐test or one‐way analysis of variance (ANOVA). GraphPad Prism v9.5 (GraphPad, Inc., USA) was used for statistical analysis. Statistical significance was set at *p* < 0.05.

## 3 RESULTS

### 3.1 NEAT1 promotes the proliferation, cell cycle progression, and EMT of cervical cancer cells

**FIGURE 1:**
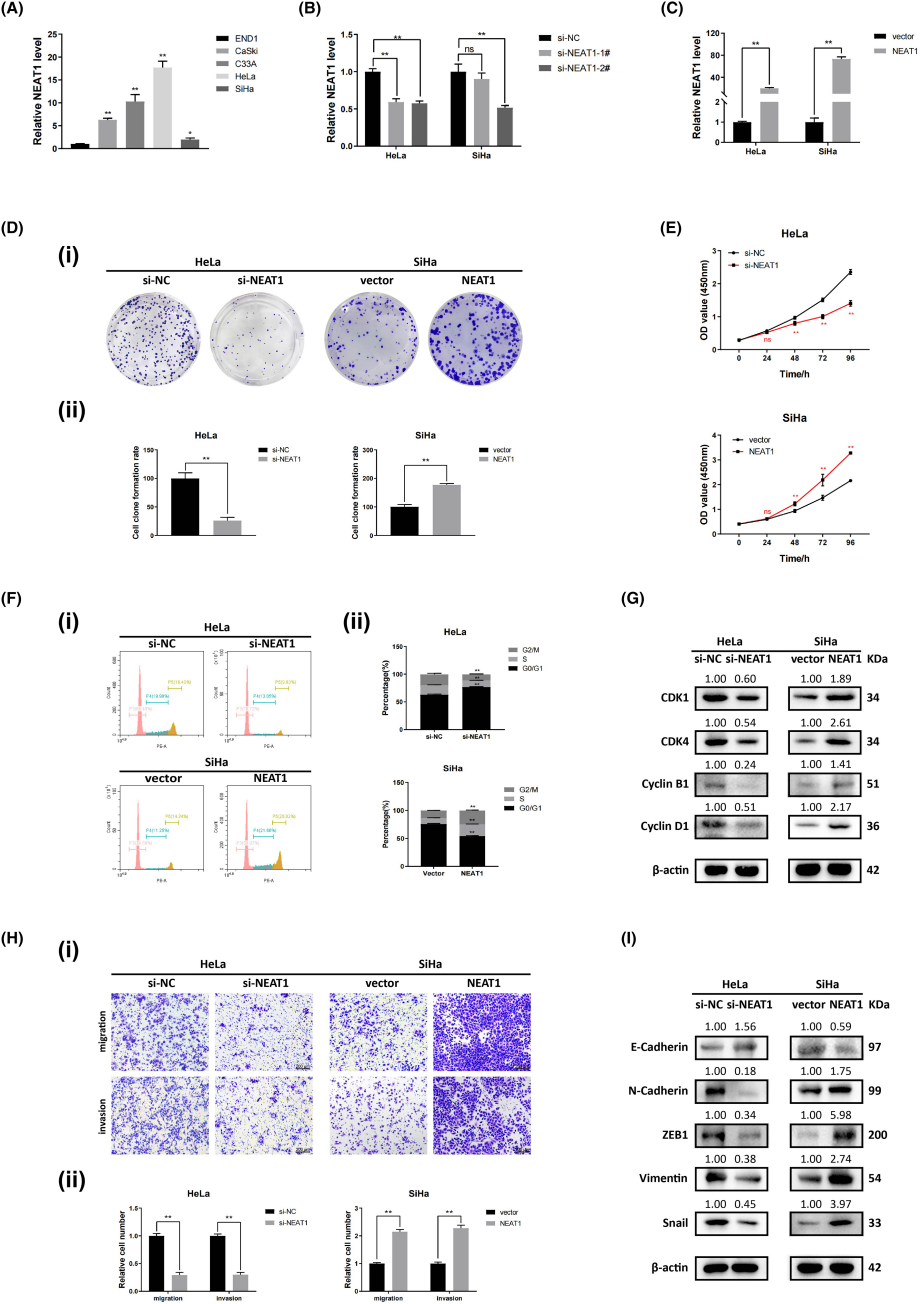
NEAT1 promotes the proliferation, migration, invasion, and EMT of cervical cancer cells. (A) Expression of NEAT1 in a human cervical squamous epithelial cell line (End1) and human cervical cancer cell lines (HeLa, Caski, C33a, SiHa). Among the four cervical cancer cell lines tested, the expression of NEAT1 was highest in the HeLa cells, and lowest in the SiHa cells. (B, C) Expression of NEAT1 in HeLa and SiHa cells transfected with siRNAs and NEAT1 overexpression plasmid, respectively. NEAT1 was significantly downregulated by siRNAs and overexpressed by plasmids. (D) CCK‐8 and (E) colony formation assays were performed to detect the proliferation ability of HeLa and SiHa cells. The proliferation of HeLa cells was suppressed after NEAT1 knockdown, while that of SiHa cells was promoted after NEAT1 overexpression. (F) The cell cycle distribution was detected by flow cytometry. (G) The expression of cell cycle‐related proteins was detected by western blotting. The cell cycle was arrested in NEAT1‐knockdown HeLa cells but promoted in NEAT1‐overexpressing SiHa cells. (H) Migration and invasion ability were detected by Transwell assays. (I) The expression of EMT‐related proteins was detected by western blotting. EMT, migration and invasion of HeLa cells were inhibited following NEAT1 knockdown, while these effects were promoted in SiHa cells following NEAT1 overexpression. The data are shown as the mean ± SD. Three individual experiments were performed. ns, *p* > 0.05; *p < 0.05; **p < 0.01; ***p < 0.001.

First, we compared NEAT1 expression in a human cervical squamous epithelial cell line (End1) and in human cervical cancer cell lines (SiHa, HeLa, C33a, Caski) using RT–qPCR. We observed that NEAT1 expression was much greater in SiHa, HeLa, C33a, Caski cell lines than in End1 cells (Figure 1A). Due to the finding that the highest expression of NEAT1 was detected in HeLa cells and the lowest expression was detected in SiHa cells among the four cervical cancer cell lines detected, we transfected HeLa and SiHa cells with two independent siRNAs targeting NEAT1 (si‐NEAT1‐1# and si‐NEAT1‐2#) and NEAT1‐expressing pcDNA3.1 plasmids to establish NEAT1‐knockdown and NEAT1‐overexpressing cell models, respectively, and confirmed the efficiency of the transfection with RT–qPCR (Figure 1B,C). NEAT1‐knockdown HeLa cells and NEAT1‐overexpressing SiHa cells were used for subsequent functional studies. Cell proliferation and colony formation were significantly suppressed after knockdown of NEAT1 in colony formation and CCK‐8 assays, while NEAT1 overexpression significantly enhanced the colony formation and proliferation of SiHa cells (Figure 1D,E). Additionally, flow cytometry analysis and western blotting analysis revealed that the proportion of cells in the G1 phase was significantly increased by NEAT1 knockdown, but NEAT1 overexpression decreased this proportion (Figure 1F). Consistently, the expression of cell cycle‐related proteins (CDK4, Cyclin D1, CDK1, and Cyclin B1) decreased in NEAT1‐knockdown HeLa cells but increased in NEAT1‐overexpressing SiHa cells (Figure 1G). We performed Transwell assays and found that the migration and invasion abilities of NEAT1‐knockdown HeLa cells were inhibited, whereas NEAT1 overexpression enhanced the migration and invasion abilities of SiHa cells (Figure 1H). As the crucial role of EMT in the migration and invasion of epithelial cell‐derived malignant tumors, we examined the expression of mesenchymal markers (N‐cadherin, ZEB1, Vimentin, Snail) and epithelial marker (E‐cadherin). The results indicated that EMT was significantly suppressed in NEAT1‐knockdown HeLa cells, while NEAT1 overexpression promoted EMT in SiHa cells (Figure 1I). These findings collectively indicate that NEAT1 enhances the growth, cell cycle progression and EMT of cervical cancer cells.

### 3.2 NEAT1 reprograms glucose metabolism in cervical cancer cells

**FIGURE 2:**
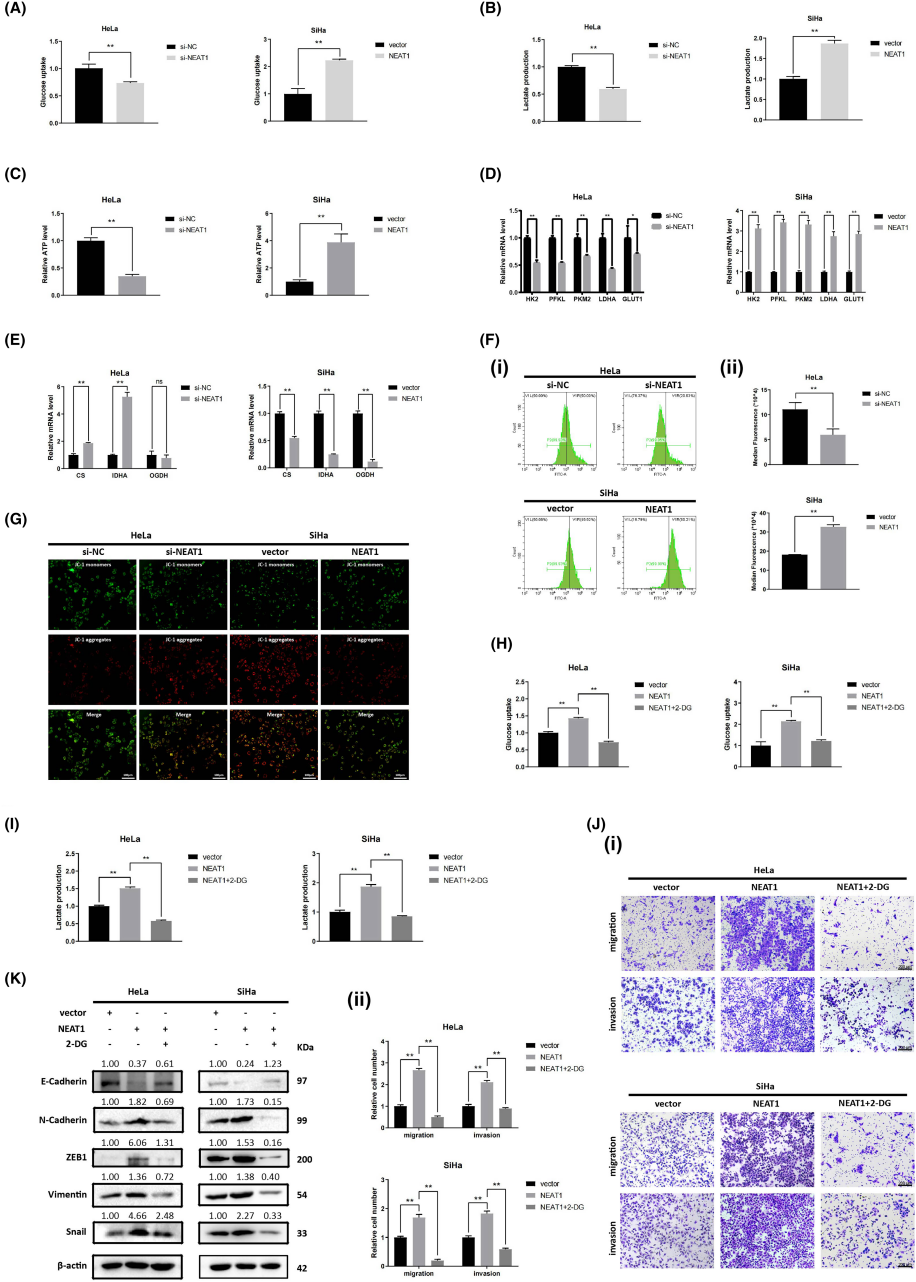
NEAT1 reprograms metabolism to promote metastasis of cervical cancer cells. (A) Glucose consumption, (B) lactate production, and (C) ATP levels in HeLa cells and SiHa cells were detected after transfection with siRNAs or NEAT1 overexpression plasmids for 48 h. Glucose consumption, lactate production and ATP levels were decreased by NEAT1 knockdown in HeLa cells but increased by NEAT1 overexpression in SiHa cells. (D) The mRNA levels of glycolysis‐related genes (HK2, PFKL, PKM2, LDHA, and GLUT1) were detected by RT–qPCR. (E) mRNA expression of tricarboxylic acid cycle‐related genes (CS, IDHA, and OGDH) was detected. The mRNA expression of HK2, PFKL, PKM2, LDHA, and GLUT1 was increased, and that of CS and IDHA was decreased in NEAT1‐knockdown HeLa cells, while NEAT1 overexpression in SiHa cells had the opposite effect. (F) ROS levels were detected with DCFH‐DA probes through flow cytometry, and representative images are shown. ROS levels were decreased in NEAT1‐knockdown HeLa cells but increased in NEAT1‐overexpressing SiHa cells. (G) The MMP was detected by JC‐1 probes through flow cytometry, and representative images were obtained under a fluorescence microscope. The MMP was increased in NEAT1‐knockdown HeLa cells but decreased in NEAT1‐overexpressing SiHa cells. (H) Glucose consumption and (I) lactate production in the control group, NEAT1‐overexpressing group and 2‐DG‐treated NEAT1‐overexpressing group were detected. 2‐DG counteracted the promotion of glucose consumption and lactate production induced by NEAT1 overexpression. (J) Transwell assays were performed to detect cell migration and invasion, and quantitative analysis of the results is shown. (K) The expression of EMT‐related proteins was detected by western blotting. 2‐DG counteracted the enhanced EMT, migration and invasion induced by NEAT1 overexpression. The data are shown as the mean ± SD. Three individual experiments were performed. ns, *p* > 0.05; *p < 0.05; **p < 0.01; ***p < 0.001.

Given the importance of metabolic reprogramming in cancer metastasis and progression, we further explored the impact of NEAT1 on the regulation of aerobic glycolysis and oxidative phosphorylation. The acidic microenvironment and ATP production caused by aerobic glycolysis play essential roles in tumor metastasis. To determine the contribution of NEAT1 to aerobic glycolysis in cervical cancer cells, we detected the levels of glucose, lactate and ATP in cervical cancer cells and found that NEAT1 knockdown led to decreased glucose consumption, lactate production as well as ATP production in HeLa cells, whereas NEAT1 overexpression led to the opposite results (Figure 2A–C). In addition, the effect of NEAT1 on the mRNA expression of glycolysis‐related genes (HK2, PFKL, PKM2, LDHA, and GLUT1) was evaluated using RT–qPCR. NEAT1 knockdown significantly downregulated the mRNA expression of glycolysis‐related genes, but NEAT1 overexpression upregulated their expression (Figure 2D). To clarify the relationship between NEAT1 and oxidative phosphorylation, we detected the mRNA expression levels of key genes involved in the tricarboxylic acid cycle (CS, IDHA, and OGDH) and found that NEAT1 knockdown in HeLa cells significantly upregulated the expression of citrate synthase (CS) and IDHA, while NEAT1 overexpression in SiHa cells significantly downregulated the expression of tricarboxylic acid cycle‐associated genes (Figure 2E). In addition, because of the iconic importance of reactive oxygen species (ROS) levels and the mitochondrial membrane potential (MMP) in mitochondrial function, we quantified ROS levels and the MMP in cervical cells with DCFH‐DA and JC‐1 fluorescent probes, respectively. NEAT1 knockdown led to reduced ROS production and an enhanced MMP, whereas NEAT1 overexpression increased ROS levels but decreased MMP levels (Figure 2F,G). Overall, we believe that NEAT1 reprograms the metabolism of cervical cancer cells, which positively regulates aerobic glycolysis and specifically inhibits mitochondrial oxidative phosphorylation.

### 3.3 NEAT1 promotes cervical cancer cell metastasis by modulating aerobic glycolysis

To verify whether NEAT1 promotes the migration and invasion of cervical cancer cells through aerobic glycolysis, the glycolysis inhibitor 2‐DG was applied to HeLa and SiHa cells overexpressing NEAT1. We observed that the enhanced lactate production and glucose consumption induced by NEAT1 overexpression were reversed after cervical cancer cells were treated with 2‐DG (Figure 2H,I). Then, the migration and invasion abilities and the expression of EMT markers in HeLa and SiHa cells were detected. The increase in migration and invasion and EMT mediated by NEAT1 overexpression were reversed following treatment with 2‐DG (Figure 2J,K). Thus, we believe that NEAT1 enhances migration and invasion abilities in cervical cancer cells by promoting aerobic glycolysis.

### 3.4 NEAT1 upregulates PDK1 to promote aerobic glycolysis in cervical cancer cells

**FIGURE 3:**
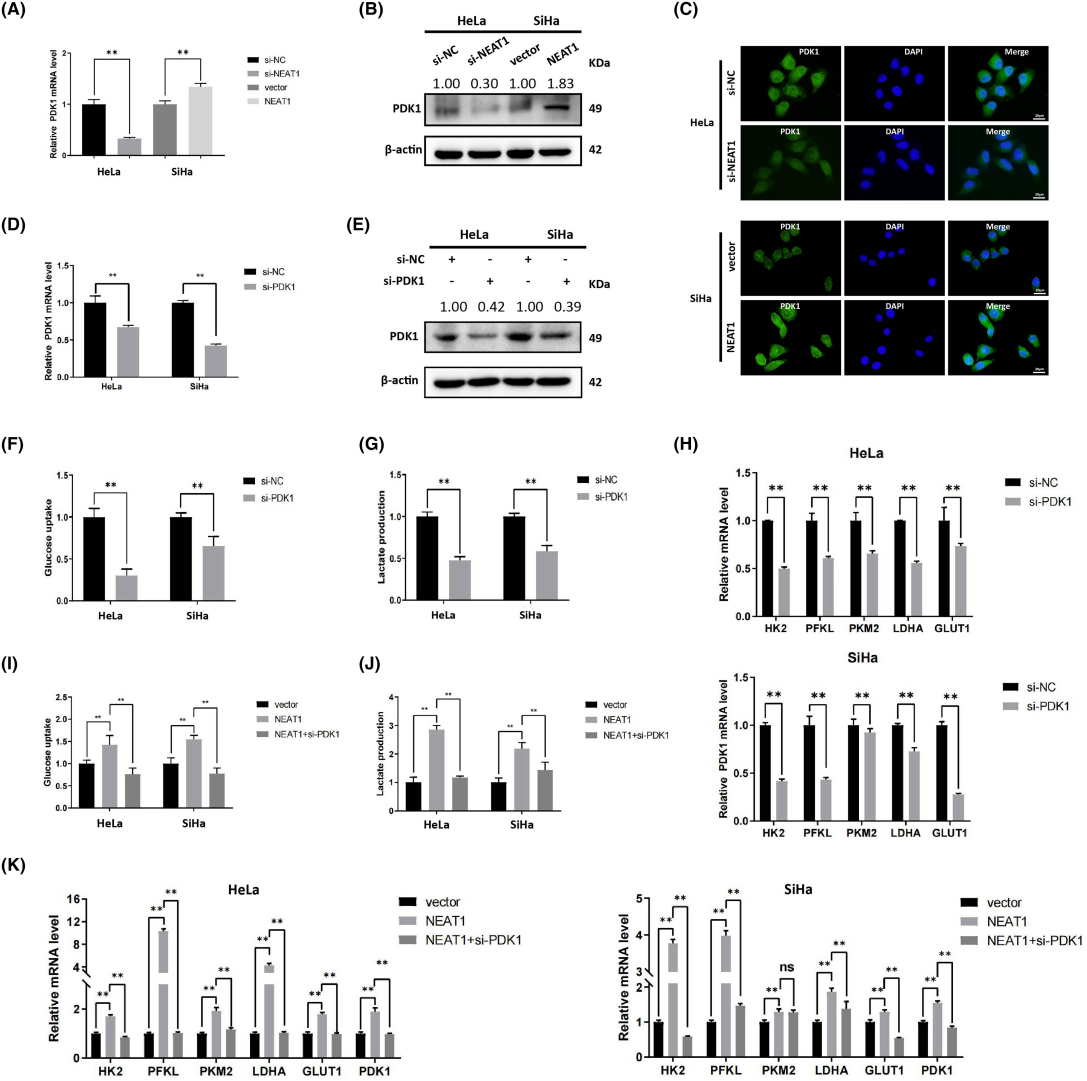
NEAT1 promotes glycolysis by upregulating PDK1. (A) The mRNA and (B) protein levels of PDK1 were detected. (C) The localization and expression of PDK1 in HeLa and SiHa cells were detected by immunofluorescence. Representative images are shown. PDK1 is expressed in both the nucleus and cytoplasm and is positively regulated by NEAT1 in HeLa and SiHa cells. (D) mRNA expression and (E) protein expression of PDK1 in HeLa and SiHa cells transfected with siRNA were detected. (F) Glucose consumption and (G) lactate production in HeLa and SiHa cells were detected. (H) The mRNA expression of glycolysis‐related genes was detected. Glucose consumption, lactate production and the mRNA expression of glycolysis‐related genes were suppressed after PDK1 knockdown in HeLa and SiHa cells. (I) Glucose consumption, (J) lactate production and mRNA expression of glycolysis‐related genes in the control group, NEAT1‐overexpressing group and NEAT1+ si‐PDK1 group were detected. PDK1 knockdown reversed the promoting effect of NEAT1 on glucose consumption, lactate production and the mRNA expression of glycolysis‐related genes. The data are shown as the mean ± SD. Three individual experiments were performed. ns, *p* > 0.05; *p < 0.05; **p < 0.01; ***p < 0.001.

PDK1 is the “gatekeeper” of glycolysis in the cell and regulates the balance between glycolysis and oxidative phosphorylation. We wondered whether PDK1 plays an essential role in the NEAT1‐mediated transition from oxidative phosphorylation to aerobic glycolysis. To this end, the PDK1 mRNA and protein were detected in NEAT1‐knockdown HeLa cells and NEAT1‐overexpressing SiHa cells (Figure 3A,B). Furthermore, the subcellular location of PDK1 was detected by an immunofluorescence assay, and we found that PDK1 was mainly located in the cytoplasm, and its expression was consistent with that of NEAT1 (Figure 3C). Moreover, PDK1 was knocked down in HeLa and SiHa cells using siRNA (si‐PDK1), and the results were verified through RT–qPCR and western blotting (Figure 3D,E). To determine the effect of PDK1 on glycolysis in cervical cancer cells, glucose consumption and lactate production, as well as the mRNA expression of glycolysis‐related genes, were detected (Figure 3F–H).

We were then interested in whether PDK1 is the crucial factor regulating the impact of NEAT1 on aerobic glycolysis in cervical cancer cells. PDK1 was knocked down using siRNA (si‐PDK1) in NEAT1‐overexpressing HeLa and SiHa cells, and glucose consumption and lactate production as well as the mRNA expression of glycolysis‐related genes were detected (Figure 3I–K). The results suggested that PDK1 knockdown significantly reversed the NEAT1 overexpression‐induced increase in glucose consumption, lactate production and glycolysis‐related gene expression in cervical cancer cells, which confirmed that NEAT1 facilitated aerobic glycolysis in cervical cancer cells by upregulating PDK1.

### 3.5 NEAT1 upregulates PDK1 through activating WNT/β‐catenin signaling pathway

#### 3.5.1 PDK1 acts downstream of WNT/β‐catenin signaling pathway

**FIGURE 4:**
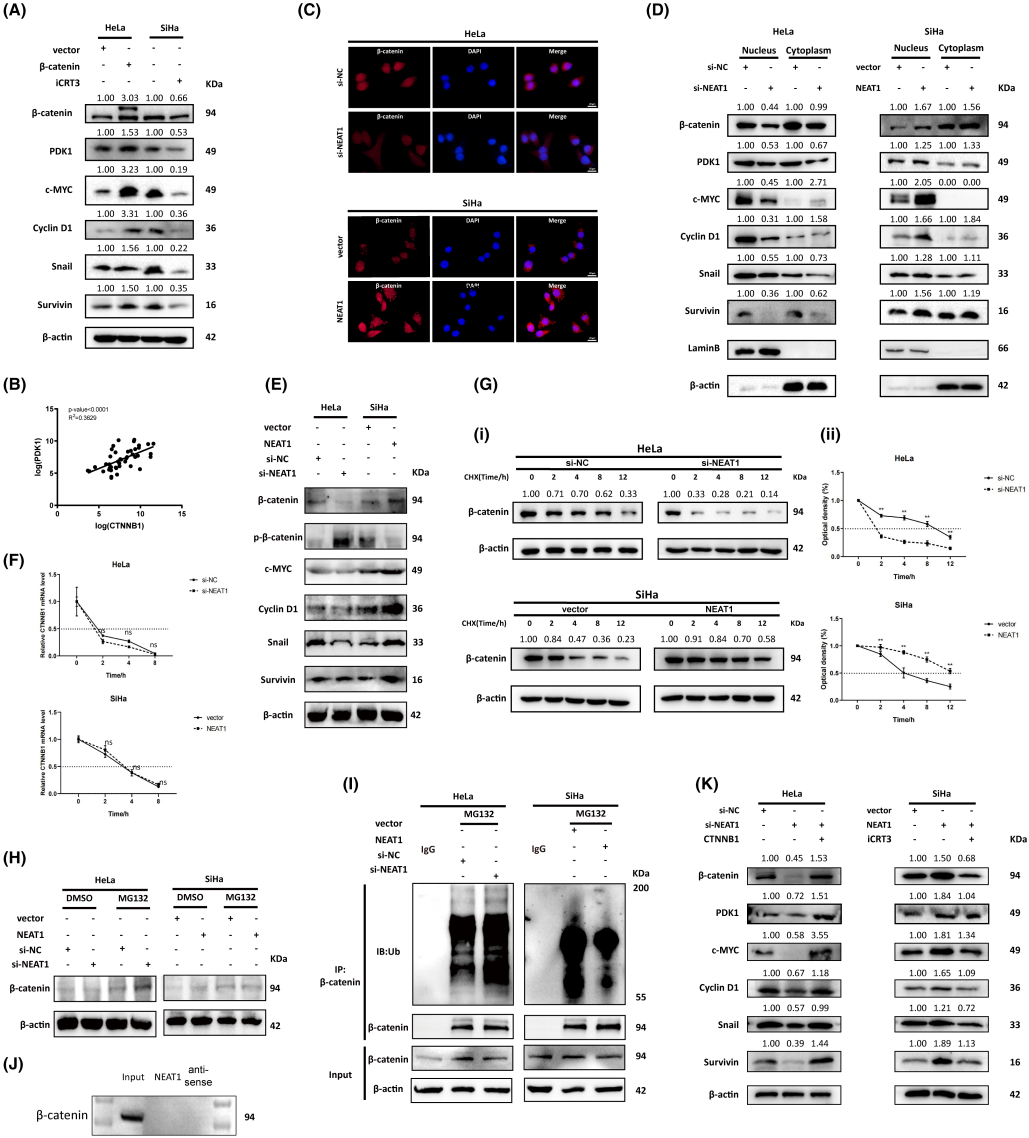
NEAT1 upregulates PDK1 by activating WNT/β‐catenin signaling pathway. (A) The expression of PDK1, β‐catenin and downstream molecules of the WNT/β‐catenin signaling pathway was detected. A CTNNB1 overexpression plasmid was used in HeLa cells to establish a WNT signaling activation model, and iCRT3 was used in SiHa cells to establish a WNT signaling inactivation model. Inactivation of WNT signaling led to a decrease in PDK1 and downstream molecules of the WNT signaling pathway, while activation of WNT signaling led to the opposite effect. (B) The correlation between the mRNA expression of PDK1 and CTNNB1 in our local cervical cancer specimens was analyzed. The mRNA expression of PDK1 was positively correlated with the mRNA expression of CTNNB1. (C) The localization of β‐catenin was detected through immunofluorescence. (D) The expression of PDK1, β‐catenin and downstream molecules in the nucleus and cytoplasm was detected. β‐catenin was located in both the nucleus and cytoplasm in HeLa and SiHa cells. After NEAT1 knockdown, nuclear β‐catenin was decreased, and cytoplasmic β‐catenin was increased. NEAT1 overexpression led to an opposite result. (E) The expression of β‐catenin, phosphorylated β‐catenin and component proteins of the WNT pathway was detected. The expression of β‐catenin and components of the WNT pathway decreased in NEAT1‐knockdown HeLa cells but increased in NEAT1‐overexpressing SiHa cells. The phosphorylation of β‐catenin was enhanced in NEAT1‐knockdown HeLa cells and reduced in NEAT1‐overexpressing SiHa cells. (F) CTNNB1 mRNA expression in HeLa and SiHa cells after treatment with Actinomycin D (Act D, 5 μM) was detected. (G) The protein expression of β‐catenin in HeLa and SiHa cells treated with CHX (20 μg/mL) was detected. NEAT1 inhibited the degradation of β‐catenin at the protein but not at the mRNA level. (H) MG132 was used in NEAT1‐knockdown HeLa cells and NEAT1‐overexpressing SiHa cells. MG132 reversed the degradation of β‐catenin caused by NEAT1 knockdown. (I) Ubiquitination of β‐catenin was detected. NEAT1 suppressed the ubiquitination of β‐catenin. (J) An RNA pull‐down assay was performed to detect the interaction between NEAT1 and β‐catenin. NEAT1 and β‐catenin cannot interact with each other. (K) The expression of PDK1 and downstream proteins of the WNT pathway was detected. CTNNB1 overexpression reversed the decrease in PDK1 caused by NEAT1 knockdown, while iCRT3 counteracted the increase in PDK1 induced by NEAT1 overexpression. The data are shown as the mean ± SD. Three individual experiments were performed. ns, *p* > 0.05; *p < 0.05; ** *p* < 0.01; ***p < 0.001.

WNT/β‐catenin signaling pathway activates PDK1 to promote aerobic glycolysis in colorectal cancer cells.^17^ Therefore, we wondered whether the enhancing effect of NEAT1 on the expression of PDK1 and aerobic glycolysis in cervical cancer cells is mediated by the WNT/β‐catenin pathway. We then transfected HeLa cells with a CTNNB1 overexpression plasmid to establish WNT/β‐catenin‐activated cervical cancer cells and used WNT signaling pathway inhibitor iCRT‐3 to inhibit WNT signaling in SiHa cells. The activity of WNT/β‐catenin pathway was confirmed and the influence of WNT/β‐catenin pathway on PDK1 expression in cervical cancer cells was measured through western blotting. Increased expression of PDK1, β‐catenin and downstream proteins of the WNT pathway was observed after overexpression of CTNNB1, whereas decreased expression was observed after inhibition of the WNT pathway (Figure 4A). In our previous study,^18^ we performed bioinformatic analysis of data from The Cancer Genome Atlas (TCGA) database and showed that the mRNA level of PDK1 was positively correlated with the expression of CTNNB1 in cervical cancer tissue. And the relationship between the mRNA expression of CTNNB1 and PDK1 was further confirmed in our local cervical cancer tissue specimens (Figure 4B). Overall, we hypothesized that PDK1 modulates aerobic glycolysis in cervical cancer cells in a WNT/β‐catenin pathway‐dependent way.

### 3.6 NEAT1 activates WNT/β‐catenin signaling pathway by maintaining the stability of β‐catenin protein

It is widely known that WNT/β‐catenin plays a material role in tumorigenesis and malignant cancer progression. Therefore, we wondered whether NEAT1 activates WNT/β‐catenin signaling pathway in cervical cancer cells. First, an immunofluorescence assay was performed to observe the location and expression of β‐catenin, a crucial component of WNT signaling pathway, in cervical cancer cells. Weaker β‐catenin expression was detected in the nucleus and cytoplasm of NEAT1‐knockdown HeLa cells, and stronger β‐catenin expression was detected in both the nucleus and cytoplasm of NEAT1‐overexpressing SiHa cells (Figure 4C). Furthermore, western blotting was conducted to investigate total protein expression and the isolated nuclear and cytoplasmic protein expression of β‐catenin, and proteins downstream of the WNT pathway. The protein expression of PDK1, β‐catenin, Snail and Survivin in both the nucleus and cytoplasm, and the expression of cyclin D1 and c‐Myc in the nucleus, decreased after NEAT1 was knocked down in HeLa cells but increased following NEAT1 overexpression in SiHa cells (Figure 4D).

Specifically, we wonder how NEAT1 changes the β‐catenin expression. We detected the expression of β‐catenin, phosphorylated β‐catenin and component proteins of the WNT pathway following NEAT1 knockdown and overexpression. We observed that NEAT1 knockdown in HeLa cells caused the downregulation of β‐catenin component proteins of the WNT pathway and the upregulation of phosphorylated β‐catenin. NEAT1 overexpression in SiHa cells had the opposite effect (Figure 4E). We wondered whether NEAT1 regulates the mRNA and protein stability of CTNNB1. Actinomycin D (Act D, 5 μM) was used to suppress the transcription of genes in cervical cancer cells, and the decrease in CTNNB1 mRNA levels was detected by RT–qPCR. However, NEAT1 had a less significant impact on the mRNA stability of CTNNB1 (Figure 4F). Moreover, cycloheximide (CHX, 20 μg/mL) was used to inhibit protein synthesis in cervical cancer cells. We observed that the expression of β‐catenin began to decline dramatically after treatment with CHX for 2 h in NEAT1‐knockdown HeLa cells, whereas the protein stability of β‐catenin was significantly enhanced in NEAT1‐overexpressing SiHa cells (Figure 4G). Furthermore, MG132 was used to inhibit protein proteasomal degradation. MG132 caused the accumulation of β‐catenin in SiHa and HeLa cells and reversed the decrease in the expression of β‐catenin induced by NEAT1 knockdown in HeLa cells (Figure 4H). Ubiquitination of β‐catenin was also detected. The results indicated that NEAT1 knockdown enhanced the ubiquitination of β‐catenin, while NEAT1 overexpression reduced it (Figure 4I). Next, we investigated whether NEAT1 interacts with β‐catenin directly to promote protein stability. We performed a RNA pull‐down assay with biotin‐labeled NEAT1 probes, but NEAT1 did not directly interact with β‐catenin (Figure 4J). These results suggested that NEAT1 maintained the protein stability of β‐catenin to activate WNT/β‐catenin signaling pathway.

### 3.7 NEAT1 upregulates PDK1 through activating WNT/β‐catenin signaling pathway

Furthermore, we detected the expression of PDK1 and downstream proteins of WNT pathway after activating the WNT/β‐catenin pathway in NEAT1‐knockdown HeLa cells or inhibiting the pathway in NEAT1‐overexpressing SiHa cells to determine whether NEAT1 regulated PDK1 through WNT/β‐catenin pathway. Western blotting revealed that CTNNB1 overexpression strikingly reversed the decrease in PDK1 expression mediated by NEAT1 knockdown in HeLa cells. In contrast, iCRT‐3 treatment reversed the increase in PDK1 expression caused by NEAT1 overexpression in SiHa cells (Figure 4K). It seems that NEAT1 regulates PDK1 expression through the WNT/β‐catenin signaling pathway.

### 3.8 NEAT1 promotes aerobic glycolysis and the progression of cervical cancer through WNT/β‐catenin signaling pathway

**FIGURE 5:**
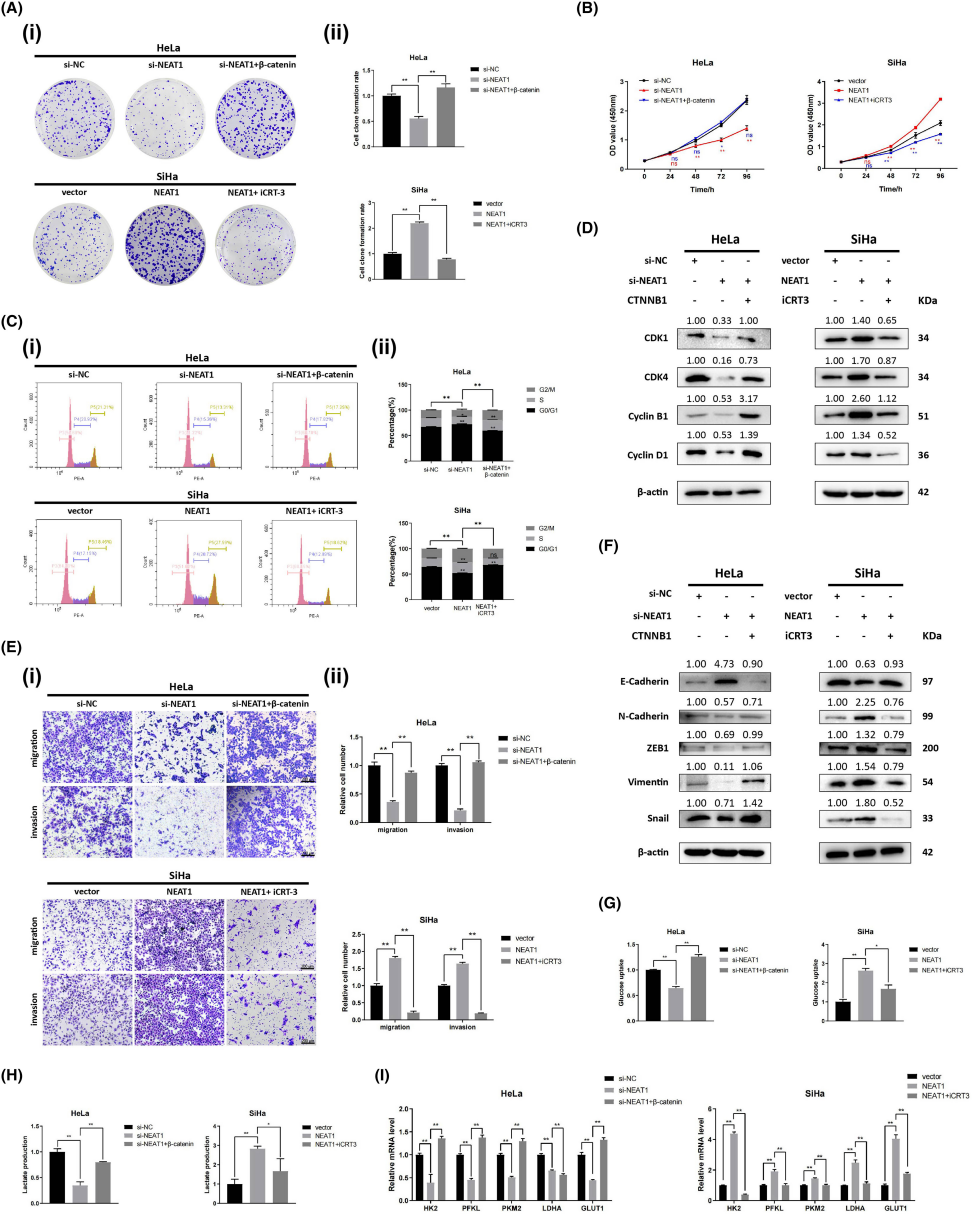
NEAT1 enhances proliferation, metastasis and glycolysis in cervical cancer cells through WNT/β‐catenin pathway. (A) Colony formation assays and (B) CCK‐8 assays were performed to detect the proliferation of HeLa and SiHa cells. CTNNB1 overexpression reversed the suppression of proliferation caused by NEAT1 knockdown, while iCRT3 counteracted the promotion of proliferation induced by NEAT1 overexpression. (C) Cell cycle analysis through flow cytometry and (D) Western blotting analyses of cell cycle‐related proteins were performed to detect the cell cycle progression of HeLa and SiHa cells. CTNNB1 overexpression reversed the cell cycle arrest mediated by NEAT1 knockdown, while iCRT3 counteracted the acceleration of the cell cycle resulting from NEAT1 overexpression. (E) Cell migration and invasion were detected by Transwell assays. (F) The expression of EMT‐related proteins was detected. CTNNB1 overexpression reversed the inhibition of migration and invasion induced by NEAT1 knockdown, while iCRT3 counteracted the enhancement of migration and invasion mediated by NEAT1 overexpression. (G) Glucose consumption, (H) lactate production and (I) mRNA expression of glycolysis‐related genes were detected. CTNNB1 overexpression reversed the suppression of glycolysis mediated by NEAT1 knockdown, while iCRT3 counteracted the promotion of glycolysis induced by NEAT1 overexpression. The data are shown as the mean ± SD. Three individual experiments were performed. ns, *p* > 0.05; *p < 0.05; **p < 0.01; ***p < 0.001.

A series of rescue assays were performed to determine the essential role of WNT/β‐catenin signaling pathway in aerobic glycolysis and the progression‐promoting function of NEAT1 in cervical cancer. Colony formation assays and CCK‐8 assays demonstrated that CTNNB1 overexpression notably reversed the decrease in growth mediated by NEAT1 knockdown in HeLa cells (Figure 5A,B). CTNNB1 overexpression reversed the NEAT1 knockdown‐induced cell cycle arrest by flow cytometry and Western blotting, respectively (Figure 5C,D). Moreover, transwell assays and the detection of EMT markers demonstrated that CTNNB1 overexpression rescued the suppression of the migration and invasion capability and EMT of HeLa cells caused by NEAT1 knockdown (Figure 5E,F). Additionally, CTNNB1 overexpression reversed the inhibition of glucose consumption and lactate production and the decrease in the mRNA expression of glycolysis‐related genes mediated by NEAT1 knockdown (Figure 5G–I). Conversely, using iCRT‐3 to inhibit the activity of WNT signaling counteracted the enhancement of proliferation, cell cycle progression, migration, invasion, EMT and aerobic glycolysis mediated by the overexpression of NEAT1 in SiHa cells, which was also reported in our previous study.^18^ In summary, this evidence suggested that NEAT1 promoted aerobic glycolysis and cervical cancer progression through WNT/β‐catenin signaling pathway.

### 3.9 NEAT1 is highly expressed in human cervical cancer tissue and is positively correlated with β‐catenin and PDK1

**FIGURE 6:**
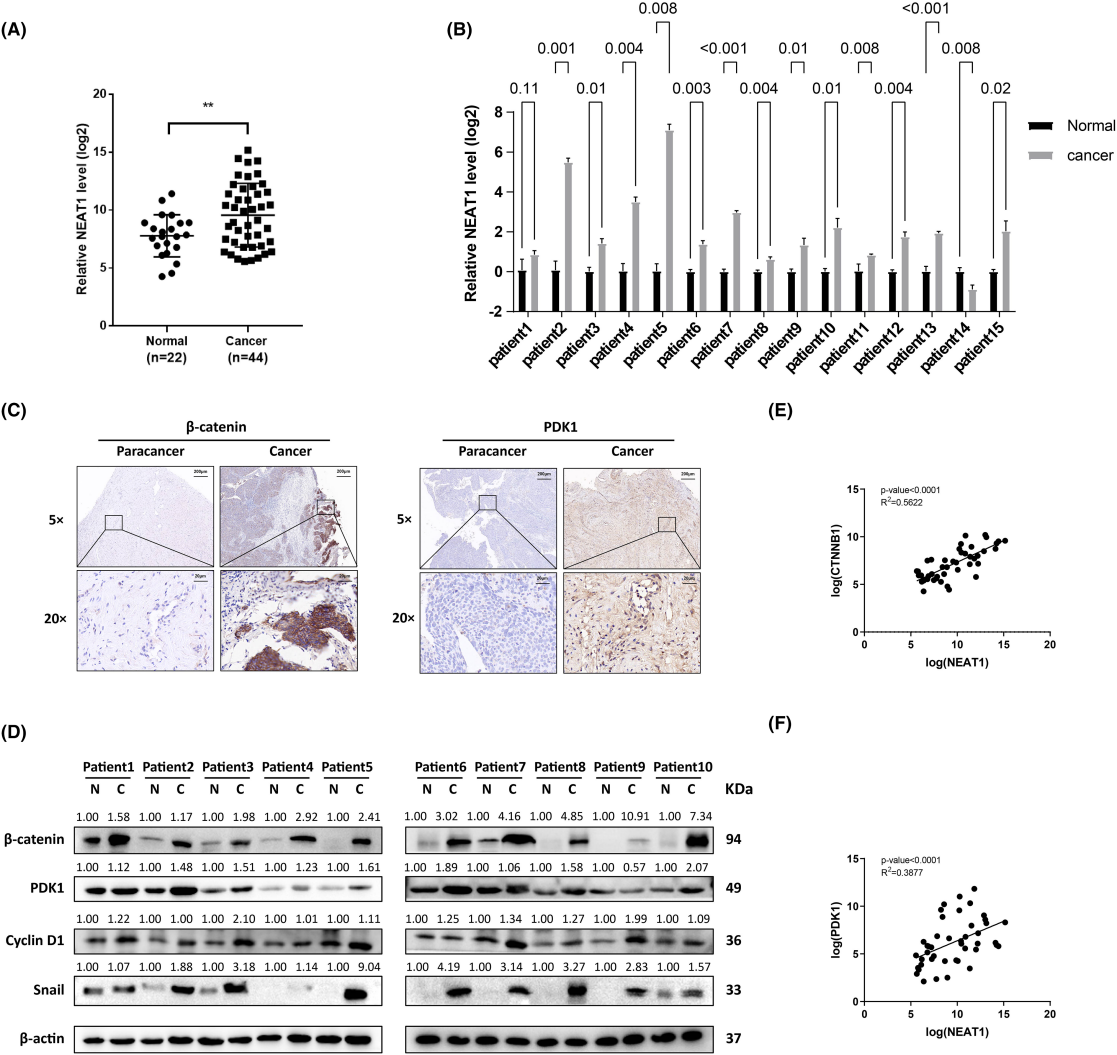
NEAT1 is highly expressed in cervical cancer tissue and is positively correlated with β‐catenin and PDK1. (A) Expression of NEAT1 in cervical cancer tissue and normal cervical tissue. (B) Expression of NEAT1 in 15 paired cervical cancer tissue and normal cervical tissue samples. Multiple t tests were used for two‐group comparisons, and the false discovery rate (FDR) method was used for correction. P‐adjust is shown on the graph. NEAT1 was highly expressed in cervical cancer tissue. (C) Expression of β‐catenin and PDK1 in cervical cancer tissue and normal cervical tissue detected by immunohistochemistry. Representative images are shown. (D) The expression of PDK1, β‐catenin and downstream molecules of the WNT/β‐catenin signaling pathway in cervical cancer tissue compared to normal cervical tissue was detected through a western blotting assay. β‐catenin and PDK1 were highly expressed in cervical cancer tissue. The WNT/β‐catenin signaling pathway was overactivated in cervical cancer tissue. (E) The correlation between the expression of NEAT1 and CTNNB1 and (F) the correlation between the expression of NEAT1 and PDK1 in cervical cancer tissue were analyzed. NEAT1 was positively correlated with the mRNA expression of CTNNB1 and PDK1. The data are shown as the mean ± SD. Three individual experiments were performed. ns, *p* > 0.05; * *p* < 0.05; ** *p* < 0.01; ***p < 0.001.

To further investigate the clinical significance of NEAT1 in cervical cancer, we compared the expression of NEAT1 between cervical cancer tissue (*n* = 44) and normal cervical tissue (*n* = 22) collected from the Affiliated Zhongnan Hospital of Wuhan University and found that compared to that in normal cervical tissue, NEAT1 expression was significantly greater in cervical cancer tissue (Figure 6A,B). A further analysis of PDK1 and β‐catenin expression in cervical cancer tissue and adjacent normal cervical tissue was completed by immunohistochemistry (IHC) and western blotting. As the results showed, PDK1 and β‐catenin were markedly more strongly expressed in cervical cancer tissue than in matched normal cervical tissue, and the downstream genes of the WNT pathway (cyclin D1, Snail) were consistently expressed according to the Western blotting analysis (Figure 6C,D). Moreover, the RT–qPCR results indicated that the mRNA expression levels of NEAT1 in cervical cancer tissue were positively correlated with the expression of PDK1 and CTNNB1 (Figure 6E,F). Overall, these results revealed that NEAT1 was highly expressed in human cervical cancer tissue and positively correlated with PDK1 and β‐catenin. Meanwhile the WNT/β‐catenin signaling pathway was overactivated in cervical cancer.

## 4 DISCUSSION

Despite the development of targeted therapy, which represents a new option for treating distant‐stage and recurrent cervical cancer,^3^ the specific mechanism underlying the progression and metastasis of cervical cancer remains unclear. Currently, substantial progress has been made in the study of lncRNAs, making people realize that lncRNAs are crucial parts of the regulation of biological processes rather than the “noise” of genome transcription.^19^ In this study, we examined the role of NEAT1 in the progression of cervical cancer and the specific mechanism underlying its function.

Glucose metabolism reprogramming allows tumor cells to gain more nutrients and biosynthetic materials, remodulating the tumor microenvironment and accelerating tumor growth. It has been proven that lncRNAs modulate aerobic glycolysis in cervical cancer cells.^20^, ^21^ Recently, the lncRNA NEAT1 was shown to regulate glycolysis to accelerate tumor progression.^22^ Specifically, NEAT1 triggers glycolysis to promote proliferation and invasion in breast cancer^23^ and enhances glycolysis by stabilizing PGK1 to promote glioma progression.^24^ In this study, we propose that NEAT1 modulates aerobic glycolysis to accelerate cervical cancer progression. PDK1 is the key “gatekeeper” in cellular glucose metabolism and inactivates PDH through phosphorylation to inhibit the tricarboxylic acid cycle in mitochondria. Pyruvate is accumulated in cytoplasm and is processed into lactate, promoting aerobic glycolysis. Our study illustrated that NEAT1 modulates aerobic glycolysis in cervical cancer by upregulating PDK1 and promoted the migration, invasion and EMT of cervical cancer cells through aerobic glycolysis.

WNT signaling pathway is involved in cellular differentiation, proliferation, metastasis and cell polarity and participates in tumorigenesis and tumor progression.^25^ The activation of classic WNT signaling pathway is characterized by accumulation and nuclear transport of β‐catenin, which binds with TCF/LEF to activate the transcription of target genes. The activation of WNT signaling has been shown to stimulate proliferation, migration, and invasion and regulate the cell cycle in cervical cancer cells.^26^, ^27^, ^28^, ^29^, ^30^ In recent years, the effect of WNT signaling on metabolic reprogramming has attracted widespread attention.^31^, ^32^, ^33^ Specifically, β‐catenin/TCF4 triggers the transcription of PKM2 to enhance the Warburg effect in colorectal cancer.^34^ Additionally, β‐catenin stabilizes HIF‐2α via interaction with HIF‐2α to upregulate aerobic glycolysis in pancreatic cancer.^35^ A previous study suggested that WNT signaling activates the transcription of PDK1 to promote aerobic glycolysis in colorectal cancer.^17^ However, whether WNT signaling modulates aerobic glycolysis in cervical cancer through regulating PDK1 remains elusive. In the present study, we demonstrated that WNT/β‐catenin signaling regulated aerobic glycolysis in cervical cancer through loss‐ and gain‐of‐expression experiments. In further exploration of the impact of the WNT/β‐catenin signaling pathway on the expression of PDK1 and the correlation between β‐catenin and PDK1, combined with bioinformatic analysis, we hypothesized that PDK1 mediates the modulation of WNT/β‐catenin signaling to aerobic glycolysis in cervical cancer.

Current studies have elucidated that NEAT1 is an essential regulatory factor of WNT signaling pathway. It has been reported that NEAT1 binding with EZH2 inhibits the transcription of the negative modulators of WNT signaling, AXIN, GSK3, and ICAT, to activate WNT signaling pathway in glioma.^36^ In addition, independent studies in endometrial cancer,^35^ colorectal cancer,^29^ small cell lung cancer^11^ and nasopharyngeal cancer^37^ have shown that NEAT1 competitively interacts with miRNAs to eliminate the inhibition of WNT/β‐catenin signaling pathway. However, the regulatory effect and specific mechanism of NEAT1 in WNT signaling remain unknown. Our study revealed that knockdown of NEAT1 suppressed the expression of β‐catenin and downstream effectors of the WNT signaling pathway and downregulated the nuclear expression and total expression of β‐catenin. Furthermore, we observed that NEAT1 enhanced the protein stabilization of β‐catenin but had no influence on the RNA stabilization of CTNNB1. NEAT1 protected β‐catenin from ubiquitination and proteasomal degradation. According to this evidence, we speculated that NEAT1 enhances the stabilization of β‐catenin protein, increasing its accumulation and transport to the nucleus to activate the WNT/β‐catenin pathway. Previous studies have reported that lncRNAs can interact with target proteins to enhance their stabilization. However, we did not observe a direct interaction between NEAT1 and β‐catenin in our study. Overall, we presumed that the promotion of cervical cancer progression by NEAT1 depends on the WNT/β‐catenin/PDK1 signaling axis.

However, further explorations should be carried out in the future. First, our study did not explore the potential regulatory effect of NEAT1 on pathways upstream of the WNT/β‐catenin pathway, such as the WNT family and destruction complexes. It remains uncertain whether NEAT1 is directly related to PDK1. Second, although the in vitro assay clearly indicated the importance of NEAT1 in the progression and aerobic glycolysis of cervical cancer, in vivo models of cervical cancer are required for a more holistic understanding of the function of NEAT1 in cervical cancer development. Third, analyses of the relationships between NEAT1 and clinicopathological parameters and patient prognosis should be performed to strengthen the clinical relevance of our findings.

**FIGURE 7:**
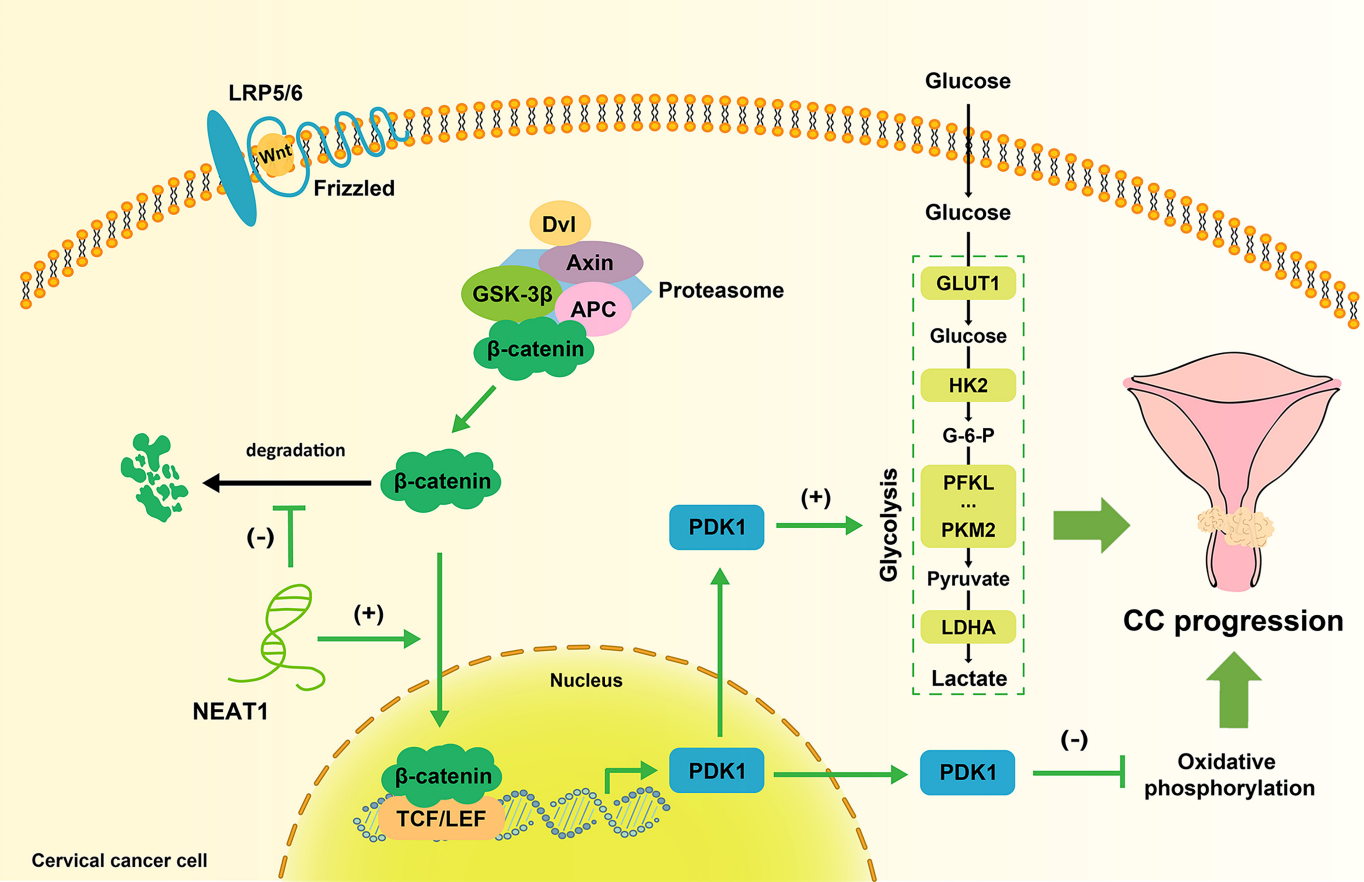
A diagram of the mechanism by which NEAT1 promotes aerobic glycolysis and cervical cancer progression via WNT/β‐catenin/PDK1 axis. In cervical cancer cells, NEAT1 upregulates the protein expression of β‐catenin by maintaining its stability and thereby activating the classical WNT/β‐catenin signaling pathway. PDK1 is upregulated downstream of WNT/β‐catenin signaling. PDK1, an enzyme that mediates glucose metabolic transformation, promotes aerobic glycolysis and inhibits oxidative phosphorylation, which leads to metabolic reprogramming and thus enhances the metastasis of cervical cancer.

In summary, our in vitro study revealed a new mechanism by which NEAT1 regulates cervical cancer progression: NEAT1 promotes aerobic glycolysis in cervical cancer cells to promote the EMT and malignant progression of cervical cancer by activating WNT/β‐catenin/PDK1 axis (Figure 7).

## AUTHOR CONTRIBUTIONS

**Min Su:** Conceptualization (equal); data curation (equal); formal analysis (equal); methodology (equal); project administration (equal); supervision (equal); visualization (equal); writing – original draft (equal); writing – review and editing (equal). **Ziyan Liang:** Data curation (equal); formal analysis (equal); investigation (equal); methodology (equal); supervision (equal); validation (equal); writing – original draft (equal). **Shidong Shan:** Data curation (equal); investigation (equal); methodology (equal); software (equal); validation (equal); visualization (equal); writing – review and editing (equal). **Yang Gao:** Conceptualization (equal); funding acquisition (equal); software (equal); visualization (equal). **Li He:** Investigation (equal); software (equal); validation (equal). **Xuelian Liu:** Data curation (equal); investigation (equal). **Anjin Wang:** Project administration (equal); visualization (equal). **Hua Wang:** Funding acquisition (equal); resources (equal); supervision (equal). **Hongbing Cai:** Conceptualization (equal); funding acquisition (equal); project administration (equal); resources (equal); validation (equal); writing – review and editing (equal).

## FUNDING INFORMATION

This work was supported by the National Natural Science Foundation of China (81972447).

## CONFLICT OF INTEREST STATEMENT

The authors have no conflicts of interest.

## ETHICS STATEMENT

Approval of the research protocol by an Institutional Reviewer Board: This study was approved by the Medical Ethics Committee of Zhongnan Hospital of Wuhan University (No. 2021087).

## INFORMED CONSENT

All informed consent was obtained from the subject.

## REGISTRY AND THE REGISTRATION NO. OF THE STUDY/TRIAL

N/A.

## ANIMAL STUDIES

N/A.

## Supporting information


Table S1.

Table S2.

Table S3.

## Data Availability

All data are available upon request.
