# Supplementary material for: Long non‐coding RNA NEAT1 promotes aerobic glycolysis and progression of cervical cancer through WNT/β‐catenin/PDK1 axis
Source: Cancer Med. 2024 May 11;13(9):e7221. doi: 10.1002/cam4.7221 (PMC11087816; doi:10.1002/cam4.7221)
Supplement: Supplementary file 1 — Table S1. [file CAM4-13-e7221-s003.docx]

| Gene | Primer sequence |
| --- | --- |
| ACTB | F: CATGTACGTTGCTATCCAGGC  R: CTCCTTAATGTCACGCACGAT |
| CTNNB1 | F: AGCTTCCAGACACGCTATCAT  R: CGGTACAACGAGCTGTTTCTAC |
| CS | F: GTCTGGCTAACACAGCTGCAGA  R: CATGGCCATAGCCTGGAACA |
| GLUT1 | F: CATCCCATGGTTCATCGTGGCTGAACT  R: GAAGTAGGTGAAGATGAAGAACAGAAC |
| HK2 | F: CCAGTTTTCCGAGAACCAAA  R: ATGCTGATCTGCTGCGTATG |
| IDH1 | F: AATCAGTGGCGGTTCTGTGGTA  R: ACTTGGTCGTTGGTGGCATC |
| LDHA | F: ATGGCAACTCTAAAGGGATCA  R: GCAACTTGCAGTTCGGGC |
| NEAT1 | F: AAACGCTGGGAGGGTACAAG  R: ATGCCCAAACTAGACCTGCC |
| OGDH | F: GGCTACGTGTTGACGCCATA  R: CTCAACTTAGCAGCACAAGTCCTTA |
| PFKL | F: GGAGAAGCTGCGCGAGGTTTAC  R: ATTGTGCCAGCATCTTCAGCATGAG |
| PDK1 | F: CGGATCAGAAACCGACACA  R: ACTGAACATTCTGGCTGGTGA |
| PKM2 | F: GAGGCTGCCATCTACCACTT  R: CACTCCTGCCAGACTTGGTG |

**Supplementary Table 1: Primers used for RT-qPCR.**
