# Supplementary material for: Long non‐coding RNA NEAT1 promotes aerobic glycolysis and progression of cervical cancer through WNT/β‐catenin/PDK1 axis
Source: Cancer Med. 2024 May 11;13(9):e7221. doi: 10.1002/cam4.7221 (PMC11087816; doi:10.1002/cam4.7221)
Supplement: Supplementary file 2 — Table S2. [file CAM4-13-e7221-s002.docx]

| Proteins | Antibody Type and Cat. No. | Reagent company | Dilution ratio |
| --- | --- | --- | --- |
| β-actin | Rabbit mAb, AC026 | ABclonal | 1:10000 |
| β-catenin | Rabbit mAb, A19657 | ABclonal | 1:1000 for Western Blotting  1:500 for IF |
| Phospho-β-Catenin-S33/S37/T41 | Rabbit pAb, AP0524 | ABclonal | 1:1000 |
| CDK1 | Rabbit mAb, ab133327 | Abcam | 1:5000 |
| CDK4 | Rabbit pAb, A21317 | ABclonal | 1:1000 |
| c-MYC | Mouse mAb, 67447-1-Ig | Proteintech | 1:2000 |
| Cyclin B1 | Rabbit pAb, 55004-1-AP | Proteintech | 1:1000 |
| Cyclin D1 | Mouse mAb, 60186-1-Ig | Proteintech | 1:2000 |
| E-Cadherin | Rabbit mAb, A22850 | Abclonal | 1:1000 |
| GAPDH | Mouse mAb, 60004-1-Ig | Proteintech | 1:5000 |
| Lamin B1 | Rabbit pAb, AF5161 | Affinity Biosciences | 1:1000 |
| N-Cadherin | Rabbit pAb, 22018-1-AP | Proteintech | 1:1000 |
| PDK1 | Rabbit pAb, A0834 | Abclonal | 1:1000 for Western Blotting  1:500 for IF |
| Snail | Rabbit pAb, A11794 | ABclonal | 1:2000 |
| Survivin | Rabbit pAb, AF0617 | Affinity Biosciences | 1:800 |
| Ubiquitin | Rabbit mAb, A19686 | ABclonal | 1:1000 |
| Vimentin | Rabbit mAb, A19607 | ABclonal | 1:800 |
| ZEB1 | Rabbit pAb, 21544-1-AP | Proteintech | 1:1000 |
| HRP-conjugated Affinipure Goat Anti-Mouse IgG(H+L) | SA00001-1 | Proteintech | 1:10000 |
| HRP-conjugated Affinipure Goat Anti-Rabbit IgG(H+L) | SA00001-2 | Proteintech | 1:10000 |

**Supplementary Table 2: Antibodies used in Western Blot experiments.**
